# Therapeutic approach of adipose-derived mesenchymal stem cells in refractory peptic ulcer

**DOI:** 10.1186/s13287-021-02584-3

**Published:** 2021-09-26

**Authors:** Mahshid Saleh, Amir Ali Sohrabpour, Mohammad Reza Mehrabi, Iman Seyhoun, Amir Abbas Vaezi

**Affiliations:** 1 Department of Applied Cell Sciences, School of Advanced Technologies in Medicine, Tehran University of Medical Sciences, Tehran, Iran; 2 Gastroenterology and Hepatology, Liver and Pancreatobiliary Diseases Research Center, Digestive Disease Research Institute, Tehran University of Medical Sciences, Tehran, Iran; 3 Department of Laboratory Sciences, Borujerd Branch, Islamic Azad University, Borujerd, Iran; 4 Department of Internal Medicine, Alborz University of Medical Science, Karaj, Iran

**Keywords:** Refractory peptic ulcer, Peptic ulcer, Mesenchymal stem cells, Adipose tissue, Cell therapy

## Abstract

Peptic ulcer is one of the most common gastrointestinal tract disorders worldwide, associated with challenges such as refractory morbidity, bleeding, interference with use of anticoagulants, and potential side effects associated with long-term use of proton pump inhibitors. A peptic ulcer is a defect in gastric or duodenal mucosa extending from muscularis mucosa to deeper layers of the stomach wall. In most cases, ulcers respond to standard treatments. However, in some people, peptic ulcer becomes resistant to conventional treatment or recurs after initially successful therapy. Therefore, new and safe treatments, including the use of stem cells, are highly favored for these patients. Adipose-derived mesenchymal stem cells are readily available in large quantities with minimal invasive intervention, and isolation of adipose-derived mesenchymal stromal stem cells (ASC) produces large amounts of stem cells, which are essential for cell-based and restorative therapies. These cells have high flexibility and can differentiate into several types of cells in vitro. This article will investigate the effects and possible mechanisms and signaling pathways of adipose tissue-derived mesenchymal stem cells in patients with refractory peptic ulcers.

## Introduction

Upper gastrointestinal tract consists of the mouth, throat, esophagus, stomach, and duodenum. The stomach is the muscular, bean-shaped part of the gastrointestinal tract meant for the initial digestion of food and infectivity [1]. The stomach eliminates incoming pathogens by secreting acidic fluids and facilitates the denaturation of proteins and the absorption of metals. Salivary amylase and peptidases secreted by corpus glands digest carbohydrates and proteins [2]. The stomach breaks down food into smaller pieces to prepare it for absorption in the small intestine. The duodenum is a 25–38 cm (10–15 inch) C-shaped structure adjacent to the stomach [3]. The incidence of ulcers increases with age for duodenal ulcers (DU) and gastric ulcers (GU). DUs, especially in men, occur two decades earlier than GU [4]. The prevalence of GU disease in the general population is estimated at 5–10% [5], and its incidence is 0.1–0.3% per year [6]. Ulcer healing is a complex, multi-step process involving inflammation, cell proliferation, re-epithelialization, angiogenesis, and other mechanisms controlled by cytokines [7, 8]. It has also been suggested that circulating ancestral cells, including bone marrow-derived stem cells, play a crucial role in healing mucous membrane ulcers, connective tissue regeneration, and neovascularization [9].

### Peptic ulcer

Peptic ulcer is caused by a defect in duodenal or gastric mucosa, and in fact, sustained activity of acid and pepsin leads to damage to the mucosa and ultimately to ulcers [10]. The outcome of peptic ulcer ranges from healing without intervention to complications such as bleeding and perforation. In the past, an acidic environment and factors such as diet or stress were thought to account for most cases of peptic ulcer; however, the discovery of *Helicobacter pylori* infection and widespread use of nonsteroidal anti-inflammatory drugs (NSAIDs) has changed this perception [5]. The incidence of ulcers has increased significantly due to extensive and long-term use of NSAIDs [11]. Therapeutic measures include medical and surgical treatments. Ulcers caused by aspirin consumption might require medical intervention and sometimes surgery. Medications involve the inhibition of gastric acid secretion by H2-receptor antagonists or proton pump inhibitors (PPI), as well as antibiotics to eliminate *H. pylori* [12]. An esophageal ulcer is an injury to the margin of the esophageal mucosa. This mucosal damage to the esophagus is often caused by gastroesophageal reflux disease or severe esophagitis caused by other factors [13, 14]. Inflammatory infections of the esophagus commonly seen in immunocompromised individuals include Candida, Herpes simplex, and Cytomegalovirus [15].

Radiotherapy is used to treat half of all cancer patients and plays a vital role in cancer treatment. The intestine is one of the most critical tissues damaged during radiation therapy of abdominal and pelvic tumors. Radiation enteropathy is generally classified into acute and chronic. Acute enteropathy occurs within 3 months after radiotherapy and chronic enteropathy for more than 3 months [16]. Acute damage from radiotherapy mainly involves rapidly proliferating cells, for example, exposing the epithelial surfaces of the skin or gastrointestinal tract. Radiation damages stem cells, and tissues are destroyed when they have an insufficient replacement with stem cells due to radiation damage [17]. Furthermore, this causes the protective barriers to disappear, especially in tissues such as the skin, oral mucosa, and gastrointestinal tract, mainly 1–5 years after the end of radiotherapy. After a while, compensatory hyperplasia in the stem cells helps the tissue to heal. When an acute injury does not fully heal, a delayed phase occurs with late consequences [18]. Such changes occur in the combination of chemotherapy and radiotherapy. The delayed effects are due to the interaction of different cytokines and cellular adaptive processes. Vascular damage leads to increased permeability and release of vascular cytokines, TGF-beta and fibrin, which cause collagen deposition [19, 20]. Chronic complications include chronic diarrhea, malabsorption, recurrent ileus attacks or obstruction, proliferative mucosal telangiectasia, or ulceration [21]. Excessive cell loss may perpetuate cytokine storms and irregular cell interactions. The type of cytokines released depends on the tissue type and is responsible for the differential response of tissues to radiation [19, 22]. Treatment strategies for chronic enteropathies include oral anti-inflammatory drugs, analgesics, stool softeners, steroid enemas, blood transfusions in the presence of bleeding, and mechanical dilatation of injuries. For severe or refractory complications, hyperbaric oxygenation, endoscopic intervention, or surgery involving colostomy may be required [23].

Although radiation therapy is effective in managing malignant tumors of the abdomen and pelvis, radiation entropy is inevitable. The disease leads to resistant lesions such as intestinal ischemia, mucositis, refractory ulcers, necrosis, or even perforation, negatively impacting the patient's quality of life [24]. A peptic ulcer is a common health problem worldwide and faces many challenges in treating resistant ulcers. In this review article, we discuss refractory peptic ulcer disease and new alternative therapies.

### Refractory peptic ulcer

A refractory peptic ulcer (RPU) is an endoscopically confirmed ulcer larger than 5 mm that does not heal after 8–12 weeks of treatment with a proton pump inhibitor. Persistent *Helicobacter pylori* can lead to refractory peptic ulcers [25] because this infection might not receive enough attention at first, *Helicobacter pylori* test is falsely negative, or the eradication treatment of this bacterium is unsuccessful [26]. In some patients, persistent ulcers may function as a severe inflammatory response, dense ulcers, or decreased mucosal blood flow impairs angiogenesis and tissue repair [27]. Peptic ulcer disease (PUD) is a significant cause for upper gastrointestinal bleeding (UGI), and *Helicobacter pylori* infection is the main causative factor of PUD. Resistant peptic ulcer disease (RPU) appears as persistent and recurrent bleeding or GI-related complications such as perforation, stenosis, and obstruction [28]. Table 1 lists various factors leading to the development of RPU [28–30]. Studies have shown that the size of an ulcer affects the healing rate so that larger ulcers need more time to heal [31]. Another condition disrupting the healing process of ulcers is the presence of fibrosis in larger ulcers [32]. In patients with refractory gastric ulcers, studying the underlying cause and risk factors in this disease is of great importance.

**Table 1:** Etiology of refractory peptic ulcers

The most common causes of refractory peptic ulcers	Less common causes of refractory peptic ulcers	Rare causes of refractory peptic ulcers
Persistent *H. pylori* infection Continued NSAID Use Medications Aspirin	Radiation therapy Crack cocaine use Mesenteric ischemia	Malignancy (gastric/lung cancer, lymphomas) Gastric acid hypersecretion (retained antrum syndrome and Zollinger–Ellison syndrome) Other viral and bacterial infections (TB, Syphilis, CMV, HPV) Sarcoidosis Behçet disease Polyarteritis nodosa Crohn disease Vasculitides Smoking Stress (acute illness, burns, head injury) Chemotherapy

The stomach and duodenum are sometimes damaged during radiotherapy for hepatocellular, biliary-pancreatic cancers, or lymphoma [33]. Radiation-refractory ulcers are challenging to treat and usually do not heal with conventional therapy for ulcers. Extracorporeal radiation and transarterial chemoembolization using radioisotopes are two treatments that can create refractory peptic ulcers [34].

### Pathophysiology

*H. pylori* infection and the consumption of NSAIDs or aspirin are the main risk factors for gastric and duodenal ulcers [6, 35, 36]. The mechanism by which *H. pylori* causes various types of lesions in gastrointestinal mucosa is not fully understood [5]. Inflammation associated with *H. pylori* infection can lead to hypochlorhydria or hyperchlorhydria and cause a specific peptic ulcer [37]. In 5–10% of patients with *H. pylori*, somatostatin decreases and gastrin increases [38]. This process is followed by increasing secretion of histamine, which leads to enhanced release of pepsin or acid from chief and parietal cells of the stomach and eventually to development of peptic ulcers [39]. NSAIDs damage the gastrointestinal mucosa through systemic and local mechanisms, but systemic inhibition of cyclooxygenase-1 (COX-1) derived prostaglandins is considered significant. Decreased prostaglandin levels are associated with reduced mucus and bicarbonate secretion, inhibiting cell proliferation and diminishes mucosal blood flow, all of which are essential for maintaining mucosal integrity [40]. Prostaglandins derived from both COX-1 and COX-2 inducible enzymes appear to play a significant role in mucosal repair [41]. The inhibition of prostaglandins, vascular damage, and local effects are involved in the pathogenesis of ulcers initiated by NSAIDs [5]. Aspirin can also cause mucosal damage through local and systemic mechanisms, albeit slightly [42].

Excessive return of acid and pepsin leads to necrosis of the superficial layers of the esophageal mucosa and causes erosion and esophageal ulcers due to gastroesophageal reflux disease. The extent of damage to the esophageal mucosa is determined by prolonged exposure to bile salts and gastric acids. Extra factors contributing to the formation of esophageal ulcers include decreased gastric acid removal from the esophagus and reduced contraction of the lower esophageal sphincter. Esophageal ulcers due to chronic gastroesophageal reflux disease are associated with decreased rest of the upper esophageal sphincter, which can accelerate laryngeal symptoms such as clearing throat, chronic cough, and sore throat [15]. Other esophageal ulcers are caused by using pills that have a low PH. These pills destroy the protective mucosal barrier and cause esophageal ulcers [43, 44].

### Mesenchymal stem cells and ulcer healing

Mesenchymal stem cells (MSC) are multipotent progenitor cells with proliferation and self-regeneration capacity [45]. To standardize MSC, the International Association of Cell Therapy (ISCT) proposes the following criteria: the ability to adhere to plastic and having a fibroblastoid phenotype, expression of CD105, CD73, and CD90 markers, non-expression of CD45, CD34, CD14, CD19, and HLA-DR surface molecules, the capacity to differentiate into cartilage, adipocyte, and bone lineages. Other features of these cells are low immunogenicity and subsequent use at the clinic in autologous and allogeneic forms [46]. MSCs, like ready soldiers, are present in all types of adult tissues, including bone marrow, adipose, skin, placenta, and heart [47]. These cells secrete various inflammatory factors and recruit inflammatory cells at the time of damage, quickly migrating through blood vessels and implanting via their surface receptors with stromal cell-derived factor (SDF-1) released from the affected area to control the immune system by secreting various factors [48, 49].

On the other hand, these cells participate in tissue repair to differentiate into various types of tissues. In addition, these cells likely play an essential role in repairing the lesion by affecting the stem cells residing in each tissue and inducing their proliferation [50]. Due to their low immunogenicity and immune-regulating properties, MSCs have therapeutic effects in various inflammatory diseases. They can implant at the injury site, limiting inflammation through the secretion of cytokines through the expression of growth factor, stimulating the healing of the ulcer, modifying host immune responses by secreting regulatory proteins and anti-apoptotic agents to alter the patient's immune system [51].

### Adipose-derived mesenchymal stem cells (AD-MSC)

ADMSCs are isolated from adipose tissue using liposuction through washing, enzymatic digestion by collagenase, centrifugation, and the isolation of ADMSCs is less invasive and more accessible than BM-MSCs [52]. ADMSCs stimulate regulatory T-cells (Tregs) and suppress Th1, Th2, and Th17 cells by increasing immune-modulatory factors such as IL-10, TGF-β, and IDO (Indoleamine 2, 3-dioxygenase). ADMSCs also downregulate inflammatory factors such as IL-4, IL-12, IL-17, tumor necrosis factor (TNF-α), interferon (IFN-γ, t-bet, CD80, CD83, and CD86) [53, 54]. ADMSCs express CD-106 marker (also known as VCAM-1), which plays a role in cellular migration at a lower level than BM-MSCs do [55]. ADMSC secrete a variety of factors, including IL-6, IL-8, interleukin-1 alpha receptor (IL-1Rα), granulocyte colony-stimulating factor (G-CSF), granulocyte–macrophage colony-stimulating factor (GM-CSF), transforming growth factor (TGF-β), prostaglandin E2 (PGE2), monocyte chemotactic protein 1 (MCP-1), nerve growth factor (NGF), and hepatocyte growth factor (HGF) [56–58].

### Therapeutic approaches for RPU and AD-MSC advantages

Therapeutic strategies for bleeding complications include endoscopic treatment, surgery, and transcatheter angiolysis embolization [28]. Medical regimens for identification and eradication of *H. pylori* and widespread use of proton pump inhibitor therapy (PPI) to suppress gastric acid secretion have led to successful management of PUD in the vast majority of patients [59, 60]. PPI is the most potent drug for the treatment of stomach ulcers. However, some gastric ulcers do not heal even with PPI treatment [29]. In these patients, anti-secretory therapies such as omeprazole are used, especially in cases of PPI resistance, which has been somewhat effective [61]. However, PPIs are the preferred treatment for patients with refractory ulcers. RPU ulcers are those gastric ulcers that do not heal entirely despite 8–12 weeks of anti-secretory drug treatment [29]. Competitive potassium acid inhibitors are also valuable for preventing NSAID-related ulcer disease, but there is no adequate access to these inhibitors [62]. Surgery is rarely performed and is only used for gastric ulcers that do not heal after treatment with anti-secretory factors and PPI for 24 weeks and are associated with conditions such as drug interactions, NSAID history, and *H. pylori* infection [63]. Recently, indications for gastric ulcer surgery include bleeding, perforation, obstruction, intractable disease, and suspected malignancy [64]. Cell-based therapies in restorative medicine have been promising for many diseases, and MSCs have been widely used in basic laboratory research up to clinical trials [65, 66]. MSCs can be effective as alternative therapies for patients due to their self-renewal, proliferation, and differentiation [67].

### Mechanism of adipose-derived mesenchymal stem cells on peptic ulcer healing

Peptic ulcer healing involves improving inflammation, cell migration and epithelial regeneration (re-epithelialization), neovascularization, glandular and matrix regeneration [9]. The process of MSCs implantation includes a cascade of interactions, the main stage of which is rolling. In this process, circulating MSCs attach to endothelial cells by adhesion molecules and then transmigrate to the damaged area [68]. Because radiotherapy-refractory peptic ulcers cause excessive cell loss and irreversible tissue damage [22, 69], an alternative cellular source to fill this gap and reconstruct tissue can benefit the patient. For this purpose, stem cells are a suitable source for repair tissue. These cell–cell contacts can be effectively improved. Mesenchymal stem cells exert their protective effect on irradiated tissue through essential mechanisms [69]. In hematopoiesis induced by mesenchymal stem cells, the cytokine storage lost after radiation, such as SDF1, GMCSF, and IL-6 is compensated by these stem cells [70]. Another mechanism is the prevention of apoptosis of hematopoietic cells and repairing bone marrow damaged by radiation [71, 72]. Radiation usually kills high-proliferation cells, including progenitor cells, and induces many changes in tissue microenvironment and endothelial cells, including apoptosis, increased permeability, and basement membrane detachment [73, 74]. In this case, the stem cells migrate to the area damaged by the radiation and secrete important cytokines and antioxidants that activate the proliferative and regenerative processes [75, 76]. One study showed that adipose-derived mesenchymal stem cells play an essential role in wound healing following radiotherapy by increasing TIMPs and TIMP1 [77].

**Fig. 1:**
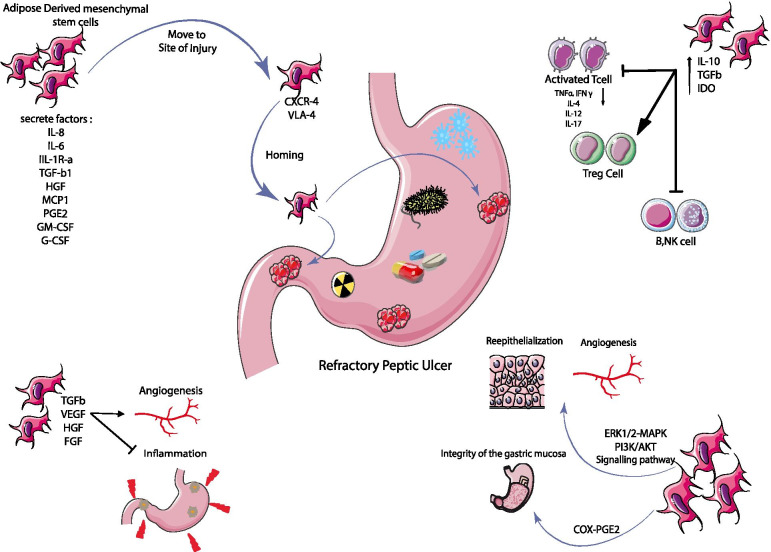
Mechanism of ADMSC cells in RPU

Several studies have shown that SDF-1 increases at the site of injury and acts as a strong adsorbent to implant MSC cells at that site [78]. Adipose-derived mesenchymal stem cells express CXCR4 and therefore migrate to the site of injury via SDF-1/CXCR4 [79, 80]. In addition, MSCs expressing the VLA-4 marker can perform the implantation process by interacting with VCAM-1 expressed on blood vessels [79, 81]. ADSCs are suitable for healing ulcers due to their ability to differentiate into several cell types, including endothelial cells and secretion of angiogenic and anti-apoptotic factors [82]. In ulcer healing, adipose tissue-derived stem cells release VEGF, TGF-β, FGF, and HGF growth factors and have beneficial effects in improving vasculogenesis and inhibiting inflammation [83–85]. Mesenchymal stem cells reduce inflammation in ulcers via secreting TGF-β [86]. They can accelerate the regeneration of colitis in laboratory models by producing hepatic growth factor (HGF), vascular endothelial growth factor (VEGF), and adiponectin [87]. VEGF secretion by ADSCs under normal and abnormal conditions has been reported as a factor for ADSC-mediated angiogenesis [88]. COX-PGE2 plays an essential role in maintaining the integrity of gastric mucosa [89]. The most likely underlying mechanism for the migration of stem cells injected through peripheral blood into damaged gastric tissue is the release of angiogenic growth factors such as VEGF and PGE2. PGE2 is an important protective factor for gastric mucosa against ulcers, healing ulcers by improving blood flow and stimulating VEGF secretion. ADSCs induce angiogenesis and heal gastric ulcers due to having VEGF and PGE2 receptors, respectively, improving blood flow and stimulating further VEGF secretion [87]. The ERK1/2-MAPK and PI3K/AKT signaling pathways are critical for cell survival, migration, and angiogenesis [90, 91]. Studies on PUD show that following injection of ADMSCs, AKT, and ERK phosphorylation increase and activate ERK1/2-MAPK and PI3K/AKT signaling pathways [89]. In gastric ulcer healing, the ERK1/2-MAPK pathway is critical for angiogenesis and re-epithelialization [90, 92]. Mesenchymal stem cells can regulate the immune response, adjusting the function of regulatory T-cells by suppressing T-cell proliferation and secretion of cytotoxic cytokines. They also inhibit the proliferation and maturation of B-cells, antigen presentation by dendritic cells, and the activation of natural killer cells by IL-2 [93]. In an environment where the IFN-γ level is elevated, MSCs enhance immunomodulatory activity by producing inflammatory inhibitors such as IDO, H6 factor, PGE-2, TGF-β, and HGF-8 [94]. The schematic mechanism of ADMSC cells in RPU is shown in Fig. 1.

Mesenchymal stem cells have been used in several animal models of gastric ulcer, some of which we will review in this study. In a study by Xianfeng et al., ADMSCs were used in an NSAID-induced peptic ulcer in a pig model. This research stated that endoscopic submucosal injection of ADMSC is a promising method in healing NSAID-related gastric ulcers and that the paracrine effects released by mesenchymal stem cells play a crucial role in this process [89]. In another study by L. Liu and colleagues, MSCs were injected locally into a mouse model with gastric perforation. This investigation showed that MSCs facilitate the improvement of gastric perforation by re-epithelialization, granulation tissue regeneration, TGF-β1 secretion, and inhibition of inflammation [95]. Another study in an indomethacin-induced gastric ulcer model showed that ADMSCs improved the histopathology of gastric tissue. In this research, 3 × 106 ADMSCs were injected through peripheral blood, and it was shown that stem cell injection reduced pathological changes in gastric ulcer morphology and restored gastric prostaglandin E2 levels to normal. Mesenchymal stem cells also increase VEGF levels to higher than usual, leading to an accelerated healing process [87]. In a study by Chang et al., BMMSCs were labeled with CFDA SE, and 1 × 107 cells were injected into a mouse model with the damaged gastric mucosa. Forty-eight and seventy-two hours after transplantation, BMMSC was present in the damaged gastric mucosa. After histopathological examination and evaluation of gastric ulcer index, they observed a significant improvement in gastric ulcer [96].

Yujiro and colleagues injected 1 × 10^7^ BM-MSC labeled with PKH67 locally into the stomach wall around the ulcer and found that MSC transplantation accelerated the healing of gastric ulcers by inducing angiogenesis in the gastric mucosa via secreting VEGF factor. The beneficial effects of these cells may be due to their differentiation into gastric interstitial cells and their ability to secrete the factors involved in angiogenesis [97]. In a study by Alazzouni et al., the anti-inflammatory effect of bone marrow mesenchymal stem cells (BM-MSCs) on piroxicam-induced gastric ulcers in rats was compared with anti-inflammatory drugs such as Pantoloc, which is a proton pump inhibitor. This study showed that BM-MSCs have a therapeutic capacity as anti-ulcers due to high antioxidant activity [98]. In another research, ADMSCs were used in aspirin-induced gastric ulcers. At 72 h after AD-MSC transplantation, there was the complete repair of the gastric fundus glands with a significant reduction in inflammatory cell infiltration. This study showed that AD-MSCs are easily isolated and removed from adipose tissue, multiplying rapidly in culture medium to play a role in the healing process of gastric ulcers [99].

## Conclusion

When RPU occurs in a patient, it is essential to have a systematic approach to diagnosis and treatment, and several treatments are available to patients. Recently, concerns have been raised about the risk of long-term use of proton pump inhibitors. For RPU-refractory and gastric ulcers, surgery is sometimes performed to reduce gastric acid secretion to heal gastric ulcers. It is unclear whether medical or surgical management is better for people with persistent or recurrent gastric ulcers. Therefore, stem cell-based therapy can be helpful in these patients. These findings are significant not only pathophysiologically but also clinically because chronic gastrointestinal ulcers are detectable and endoscopic. Therefore, MSC transplantation can be considered a new therapeutic strategy for resistant gastrointestinal ulcers. Moreover, radiation enteropathy is a frequent complication in patients with abdominal malignancy, but no treatment has yet been identified. Mesenchymal stem cell therapy may be a valuable treatment option for radiation-resistant ulcers because the loss of stem cell tissue explains the incurability of these diseases.

## Data Availability

Not applicable.

## Abbreviations

MSCs: Mesenchymal stem cells
ASC: Adipose-derived mesenchymal stromal stem cells
AD-MSC: Adipose-derived mesenchymal stem cells
BM-MSCs: Mone marrow mesenchymal stem cells
DU: Duodenal ulcers
GU: Gastric ulcers
NSAIDs: Nonsteroidal anti-inflammatory drugs
GU: Gastric ulcer
PPI: Proton pump inhibitors
RPU: Refractory peptic ulcer
PUD: Peptic ulcer disease
UGI: Upper gastrointestinal bleeding
COX-1: Cyclooxygenase-1
ISCT: International Society for Cell Therapy
VEGF: Vascular endothelial growth factor
VCAM-1: Vascular cell adhesion molecule 1
PGE2: Prostaglandin E2
bFGF: Basic fibroblast growth factor
NGF: Nerve growth factor
TGF-b1: Transforming growth beta-1
IFN-γ: Interferon-γ
TNF-α: Tumor necrosis factor α
IL-1α: Interleukin-1 alpha
SDF-1: Stromal cell-derived factor 1
MCP 1/CCL2: Monocyte chemoattractant protein-1
HGF: Hepatocyte growth factor
G-CSF: Granulocyte-colony stimulating factor
GM-CSF: Granulocyte–macrophage colony stimulating factor
Tregs: Regulatory T-cells
VLA-4: Very late antigen 4
